# Gut Microbiota Dysbiosis and Altered Bile Acid Catabolism Lead to Metabolic Disorder in Psoriasis Mice

**DOI:** 10.3389/fmicb.2022.853566

**Published:** 2022-04-14

**Authors:** Yan Hao, Pei Zhou, Ya-juan Zhu, Song Zou, Qixiang Zhao, Jiadong Yu, Yawen Hu, Jiong Li

**Affiliations:** ^1^State Key Laboratory of Biotherapy and Cancer Center, West China Hospital, West China Medical School, Sichuan University, and Collaborative Innovation Center for Biotherapy, Chengdu, China; ^2^Department of Biotherapy and Cancer Center, State Key Laboratory of Biotherapy, West China Hospital, Sichuan University, Chengdu, China; ^3^Department of Cardiology West China Hospital, Sichuan University, Chengdu, China

**Keywords:** Psoriasis, metabolic disorder, gut microbiota, fecal transfer, bile acid

## Abstract

Patients with psoriasis tend to have significant comorbidities, such as hyperlipemia, diabetes mellitus, and obesity, which belong to metabolic disorders. The specific mechanism through which psoriasis increases the metabolic disorder risk is uncertain. In this study, we demonstrated that the dysbiotic gut microbiota of 6-month-old psoriasis-like model mice (K14-VEGF-A-transgenic) exacerbated psoriasis disease and induced metabolic disorder when transferred into 2-month-old mice. By 16S rRNA gene sequencing, we confirmed that the *Parabacteroides distasonis* decreased with age in K14-VEGF mice, and *P. distasonis* also decreased in the transferred mice. Metabolomic screening identified an altered bile acid profile, including a decrease in chenodeoxycholic acid (CDCA) in the feces of transferred mice. Additionally, CDCA supplements prevented metabolic disorders in K14-VEGF-A-transgenic mice. Consequently, we found that aberrant bile acid metabolism may contribute to metabolic disorder in K14-VEGF-A-transgenic mice, indicating the possibility to prevent and treat the metabolic disorder in psoriasis mice by targeting gut microbial metabolites.

## Introduction

Psoriasis, a chronic and immune-mediated skin disease, has an estimated global prevalence of 2–3% (Lebwohl, 2003). It is associated with multiple comorbidities (Boehncke and Schon, 2015). Studies have demonstrated that individuals with psoriasis are at an increased risk of metabolic disorders, such as dyslipidemia, diabetes mellitus, obesity, and non-alcoholic fatty liver disease (NAFLD) (Khalid et al., 2013; Candia et al., 2015; Lonnberg et al., 2016; Rutter et al., 2016; Rodriguez-Zuniga and Garcia-Perdomo, 2017; Takeshita et al., 2017; Gui et al., 2018; Holmannova et al., 2020). In particular, metabolic comorbidities induce a greater risk of severe vascular diseases in patients with psoriasis. It directly increases the premature mortality in patients with psoriasis, thus substantially reducing their life expectancy (Malik et al., 2004; Mottillo et al., 2010; Manolis et al., 2019). However, the underlying mechanisms between psoriasis and metabolic disorders remain largely unknown. It is difficult to prevent and efficiently treat the metabolic disease in patients with psoriasis, highlighting the urgent need to understand the pathogenesis of metabolic disorder disease.

Trillions of microbes inhabit the human gastrointestinal tract, and numerous studies have suggested that this ecosystem significantly contributes to the host physiology by affecting host metabolism (Sommer and Backhed, 2013). In fact, several animal and human studies have reported that gut microbiota change significantly with age (Cheng et al., 2013), and a specific alteration of gut microbiota configurations promotes the development of metabolic diseases (Wu et al., 2015). Hence, gut microbiota dysbiosis is closely related to host metabolic homeostasis. Microbial fermentation of the colon yields a great diversity of metabolites, which are most often considered for regulating gut barrier integrity and metabolic health (Zhao et al., 2018; Canfora et al., 2019). For instance, microorganisms can regulate host metabolism and immunity *via* their abundant metabolites, such as short-chain fatty acids (SCFAs) and bile acids (Fan and Pedersen, 2021). Gut microbiota produce numerous metabolites, some of which are absorbed into the systemic circulation and are biologically activated, whereas others are further metabolized by host enzymes and then mediate effects of microbiota on the host (Wang et al., 2011; Koeth et al., 2013). These mediators are considered key metabolic regulators that can target different organs and ultimately regulate a range of functions in a beneficial or harmful way (Cani, 2019). Thus, abnormal composition and/or functions of the gut microbiota might contribute to a disturbed energy and substrate metabolism of adipose tissue and organ, such as muscle and liver (Le Chatelier et al., 2013).

It has been reported that the gut microbiome composition is different between patients with psoriasis and healthy individuals, and the gut microbiota profile of patients with psoriasis displays a clear dysbiosis (Codoner et al., 2018; Hidalgo-Cantabrana et al., 2019; Dei-Cas et al., 2020). However, in patients with psoriasis, a causal relationship between the gut microbiome and the development of metabolic disorders has not been firmly established. In this study, we performed animal studies to explore the role of gut microbiota and metabolites in the metabolic disorder of psoriasis. We intended to provide new evidence for the emergence of the gut microbiome in the metabolic diseases of psoriasis.

The keratin 14 (K14)-vascular endothelial growth factor A (VEGF-A)-transgenic mouse model, a psoriasis animal model that overexpresses VEGF in the epidermis, can spontaneously develop a chronic inflammatory skin disease similar to human psoriasis (Xia et al., 2003). In our study, to investigate whether the gut microbiota of psoriasis mice is related to metabolic disorders, we detected the alteration of metabolic status, gut microbiota composition, and bacterial-associated metabolites of 2-, 4-, and 6-month-old K14-VEGF-A-transgenic mice. Then, we observed that metabolic disorder was accelerated in 2-month-old mice after the feces of 6-month-old mice were transferred to 2-month-old mice. We also analyzed the fecal microbiome and fecal metabolites to find the insightful clues revealing the pathogenesis of metabolic diseases in psoriasis.

## Methods

### Animals

The K14-VEGF transgenic homozygous male mice were purchased from Jackson Laboratories (Bar Harbor, ME, USA). Genotyping of K14-VEGF mice was performed with genomic DNA extracted from the tail using the Mouse Direct PCR Kit (Bimake, B40015). The K14-VEGF transgenic mice are based on the FVB genetic background. A murine cDNA coding for VEGF-A164 was ligated into a cassette containing the human K14 promoter. The resulting construct was then injected into FVB/N zygotes. In our study, we use K14-VEGF mice as our psoriasis model, and use FVB as healthy control. Mice were used in accordance with National Institutes of Health guidelines for animal care. The animal protocol was approved by the Institutional Animal Care and Treatment Committee of Sichuan University (Chengdu, PR China). The animals were housed at a 12 h light/12 h dark cycle, a constant temperature of 25 ± 1°C, and with free access to water and food. Every effort was made to decrease the number of animals used and to reduce animal suffering.

### Fecal Microbiota Transplant

Two-month-old K14-VEGF mice were randomized into two groups, namely, fecal recipient and control. The donor group consisted of 6-month-old K14-VEGF mice. Three groups were raised in separate sterile cages. All mice were treated with a cocktail of antibiotics [AMNV: ampicillin (1 g/L), metronidazole (1 g/L), neomycin (0.5 g/L), and vancomycin (0.5 g/L)] for 5 days. Fresh fecal pellets from 6-month-old K14-VEGF mice were collected in sterile tubes. The fecal pellets were placed on the ice during the collection and then stored at −80°C. The pooled fecal pellets were diluted in sterile phosphate-buffered saline (PBS) (one pellet per 150 μl) and centrifuged at 500 g for 3 min to take the supernatant. Each recipient mouse was gavaged with 200 μl of fecal supernatant. Controls only received isovolumetric PBS. Each group was gavaged once a week until the mice were sacrificed. Phenotypes were evaluated 8 weeks after the transfer. A schematic diagram of our study is described in Supplementary Figure 1.

### Psoriasis Severity Evaluation

The clinical psoriasis area and severity index (PASI) was used to assess the severity of ear lesions. The erythema, scaling, and thickening were scored separately with a scale from 0 to 4 as follows: 0, none; 1, slight; 2, moderate; 3, serve; 4, very serve. The total score (0–12) was used to measure the ear lesions severity (van der Fits et al., 2009).

### Hematoxylin and Eosin (H&E) Staining

Ears and livers of K14-VEGF mice were fixed in 4% paraformaldehyde, embedded in paraffin, sectioned, and stained with H&E for histopathological examination. Olympus BX600 microscope (Olympus Corporation, Tokyo, Japan) and SPOT Flex camera (Olympus Corporation, Tokyo, Japan) were used to capture images. Images were analyzed using Image-Pro Plus (version 6.0, Media Cybernetics) software. According to the Baker score (Baker et al., 1992; Ren et al., 2009) system, the pathological score of a mouse ear was obtained to assess the psoriasis severity. Each liver section was graded based on a steatosis score. The scope of steatosis was scored as 0 (no steatosis), 1 (<25% of hepatocytes), 2 (26–50%), 3 (51–75%), and 4 (>75%) (Shi et al., 2010; Zhang et al., 2020). The mean epidermal thickness and pathological score were obtained in 5 random fields (400×) of each H&E-stained section.

### Oil Red O Staining

After being euthanized, the liver tissue was immediately removed and immersed in precooled PBS, fixed in 4% paraformaldehyde for 48 h. Then, the samples were incubated in a liquid nitrogen flash freezer and embedded in Tissue-Tek O.C.T. Compound (Sakura Finetek USA). Tissues were sliced into 8-mm thickness. Sections were stained with 0.5% Oil Red O (0.5 g dissolved in 100 ml isopropyl alcohol) for 10 min, counterstained with hematoxylin. The red lipid droplets were visualized using microscopy.

### Serum Biochemical Indices Analysis

To determine the serum lipid levels, each group was fasted overnight and then refed for 4 h. The mice were euthanized and their blood was collected. Total triglyceride (TG), high-density lipoprotein (HDL), low-density lipoprotein (LDL), aspartate aminotransferase (AST), and alanine aminotransferase (ALT) levels were measured *via* Biochemistry Analyzer (Roche, cobas 6000 c501/cobas c311).

### Blood Glucose Measurement

An intraperitoneal glucose tolerance test (IPGTT) was performed in each group. Mice were weighed and then fasted for 6 h from 9:00 a.m. An initial blood sample was taken at 0 min *via* acupuncture of the tail tip. Then, mice were intraperitoneally injected with glucose (Sigma, USA), containing 2 mg of glucose per gram. Subsequently, after the gentle puncture of the tail tip, a fresh spot of blood was taken at 15, 30, 60, 90, and 120 min, respectively. The blood samples were directly transferred from the tails onto an ACCU-CHEK Performa test strip (Roche, US). Blood glucose was read by an ACCU-CHEK Performa (Roche Diagnostics, USA).

### Gut Microbiota Analysis

Fecal samples were stored at −80°C, and some of them were processed for microbiota analysis by Beijing Genomics Institute, Inc. The MoBio PowerSoil Kit (MoBio, USA) was used to extract bacterial genomic DNA from fecal samples. The V4–V5 regions of the 16S rRNA gene were amplified by individually barcoded universal primers. The purified PCR products were pooled and sequenced on the HiSeq Illumina 2500 platform. Based on 97% sequence similarity to the Greengene database, operational taxonomic units (OTUs) were chosen by open reference OTU picking. Taxonomy assignment and rarefaction were conducted *via* QIIME1.8.0. Principal coordinate analysis (PCoA) and alpha diversity analyses, including Chao's richness, were performed using QIIME. Linear discriminant analysis effect size (LEfSe) analyses were performed to identify significantly enriched microbial species. In LEfSe analyses, taxa with values [*P* < 0.05 by the Kruskal–Wallis test; linear discriminant analysis (LDA) score >4] were considered statistically significant to plot a cladogram on the basis of their phylogenetic relationship.

### Metabolomic Analysis

Mice were fasted for 12 h and then fed for 4 h. Then, fecal samples were collected, immediately frozen in liquid nitrogen, and stored at −80°C. Sample preparation and analysis were performed using the liquid chromatography (LC)/mass spectrometry (MS) metabolomic platforms of Beijing Genomics Institute, Inc. Briefly, 100 μl samples were extracted by directly adding 300 μl of precooled methanol and acetonitrile (2:1). Internal standard mix 1 (IS1) and IS2 were added to control sample preparation quality. After vortexing for 1 min and incubation at −20°C for 2 h, samples were centrifuged (20 min, 4,000 rpm). Then the supernatant was transferred to vacuum freeze-drying. Samples were resuspended in 150 μl of 50% methanol and centrifuged (30 min, 4,000 rpm), and the supernatants were collected for the LC/MS analysis. The same volume of each sample was mixed as quality control to assess the reproducibility of the whole analysis. Samples were analyzed on a Waters 2D UPLC (Waters, USA), coupled to a Q-Exactive mass spectrometer (Thermo Fisher Scientific, USA) and controlled by the Xcalibur 2.3 software program (Thermo Fisher Scientific, Waltham, MA, USA). Waters ACQUITY UPLC BEH C18 column (Waters, USA) was used to conduct chromatographic separation. Principal component analysis and hierarchical clustering were performed using R.

### Drug Treatment During CDCA Gavage

Three-month-old K14-VEGF male mice were divided into two groups. One group was given chenodeoxycholic acid (CDCA) (K14-CDCA group) every day by gavage administration. CDCA (Sigma, USA) was dissolved in normal saline at 10 mg/kg and sodium carboxymethyl cellulose was used as a suspension agent (1%). The control group (K14-control) was gavaged with solvent.

### Quantification and Statistics Analysis

The hepatocyte surface Oil Red staining area quantifications were conducted using ImageJ. Statistical analyses and graphs were performed *via* the GraphPad Prism 8.0 software. A two-tailed *t*-test determined the statistical significance of the two groups. A one-way ANOVA comparative analysis was conducted among the three groups. Two-way ANOVA analysis compared the multifactor differences among the three groups. All data were expressed as mean ± standard error. Statistical significance is represented by ^*^P < 0.05, ^**^P < 0.01, ^***^P < 0.001, and ^****^P < 0.0001.

## Results

### The Metabolic Disorder Risk Increased in Aged K14-VEGF-A-Transgenic Mice Compared to Aged-Matched FVB

To investigate whether the K14-VEGF-A-transgenic psoriasis mouse model aggravated psoriasis-like dermatitis with age, we used 2-, 4-, and 6-month-old K14-VEGF-A-transgenic mice to observe the severity of psoriasis-like dermatitis. The epidermis of 2-month-old mice had no obvious psoriasis-like features. A weak but observable dermatitis was displayed in 4-month-old mice, while a typical clinical and pathological alteration of psoriasis-like dermatitis as indicated by the scores of skin scaling, erythema, hardness, and thickness was observed in 6-month-old mice (Supplementary Figures 2A–C). These data suggested that pronounced skin lesions characterized by erythematous and scaly skin are exacerbated with age, consistent with a previous study (Xia et al., 2003).

**Figure 1:**
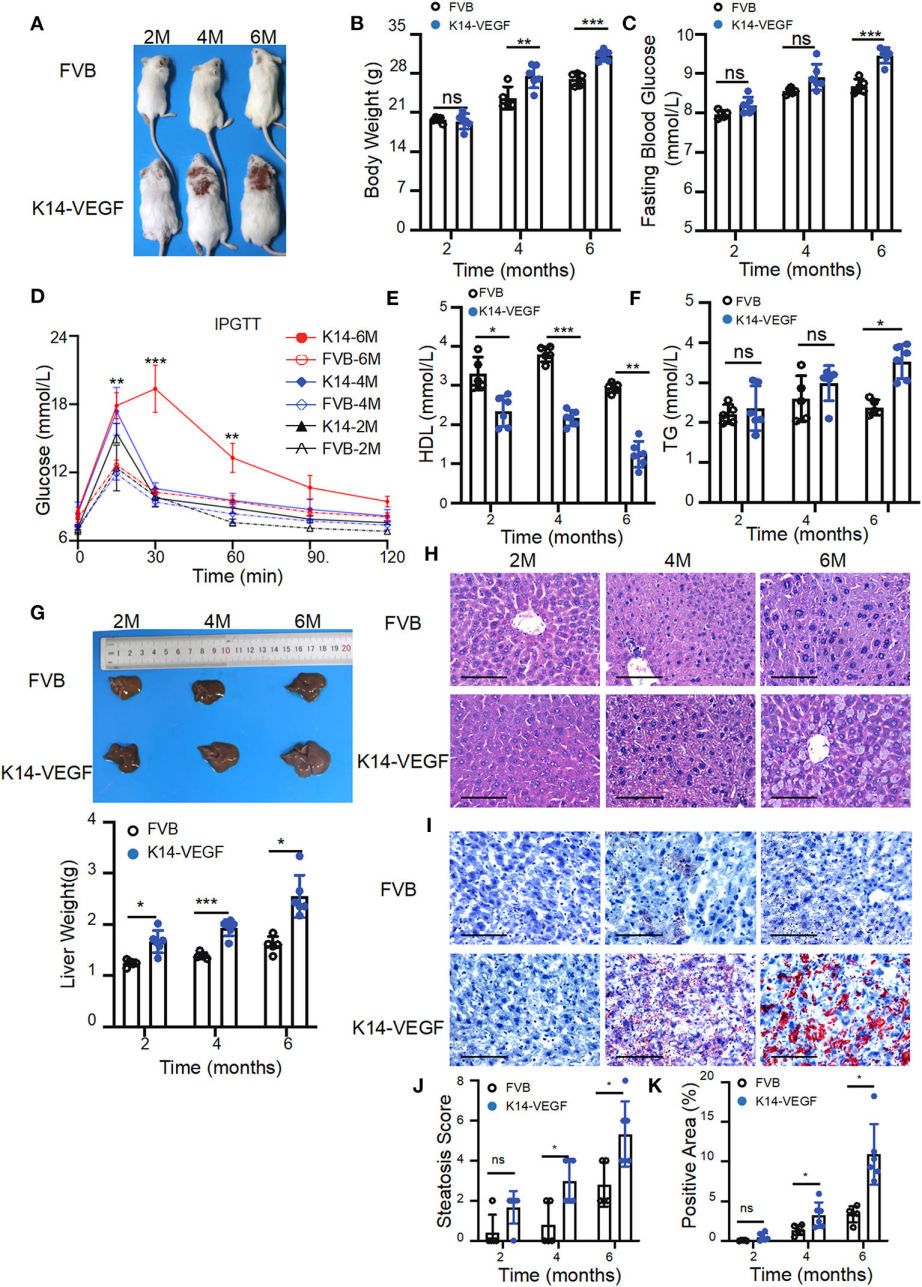
Aged K14-VEGF-A-transgenic mice, which exhibited psoriasis-associated phenotypes were more likely to have metabolic disorders compared to FVB. **(A)** Representative images of each group. FVB (*n* = 5) and 2-, 4-, and 6-month-old K14-VEGF**-**A-transgenic mice (*n* = 6). **(B)** Body weight of mice. **(C)** Fasting serum glucose levels. **(D)** Blood glucose concentrations following intraperitoneal glucose tolerance tests. **(E)** TG levels. **(F)** HDL levels. **(G)** Representative pictures and weight of livers. **(H)** Representative H&E-stained sections of liver. Scale bar, 100 μm. **(J)** Degrees of hepatic steatosis. **(I)** Oil red Staining of livers. Scale bar, 100 μm. **(K)** Quantification of hepatic ORO. Statistical analysis for **(B**–**K)**: Each symbol represents a mouse; bars show means and SEM. Statistical tests used were two-way ANOVA in all data. *P < 0.05, **P < 0.01, and ***P < 0.001.

To verify whether K14-VEGF-A transgenic mice are more prone to metabolic disorders than FVB mice, here, we compared the metabolic indexes of FVB mice and 2-, 4-, and 6-month-old K14-VEGF-A-transgenic mice. We found that the body weight of K14-VEGF-A-transgenic mice was significantly higher compared with that of 4 and 6-month-old FVB mice (Figures 1A,B). Furthermore, the fasting blood glucose levels of K14-VEGF-A-transgenic mice were higher than those of FVB mice (Figure 1C). Moreover, glucose tolerance as determined by the glucose tolerance test (GTT) was obviously attenuated in K14-VEGF-A-transgenic mice compared with FVB (Figure 1D). The serum TG levels of K14-VEGF-A-transgenic mice were significantly higher than those of FVB mice, whereas HDL levels were lower than that of FVB mice (Figures 1E,F). In addition, compared with age-matched FVB mice, K14-VEGF-A-transgenic mice exhibited increased hepatomegaly and liver weight with age increasing (Figure 1G), simultaneously accompanying with the presence of features non-alcoholic fatty liver disease (NAFLD), including liver cell hypertrophy and accumulation of lipid droplets in the liver (Figures 1H–K). K14-VEGF-A-transgenic mice showed no difference in hepatic fibrosis, ALT, and AST levels (Supplementary Figures 3A–C). These results indicated that K14-VEGF-A-transgenic mice had a higher risk of metabolic disorder than FVB mice with age increasing.

### Bacterial Communities Altered in 2-, 4-, and 6-Month-Old K14-VEGF-A-Transgenic Mice Feces

**Figure 2:**
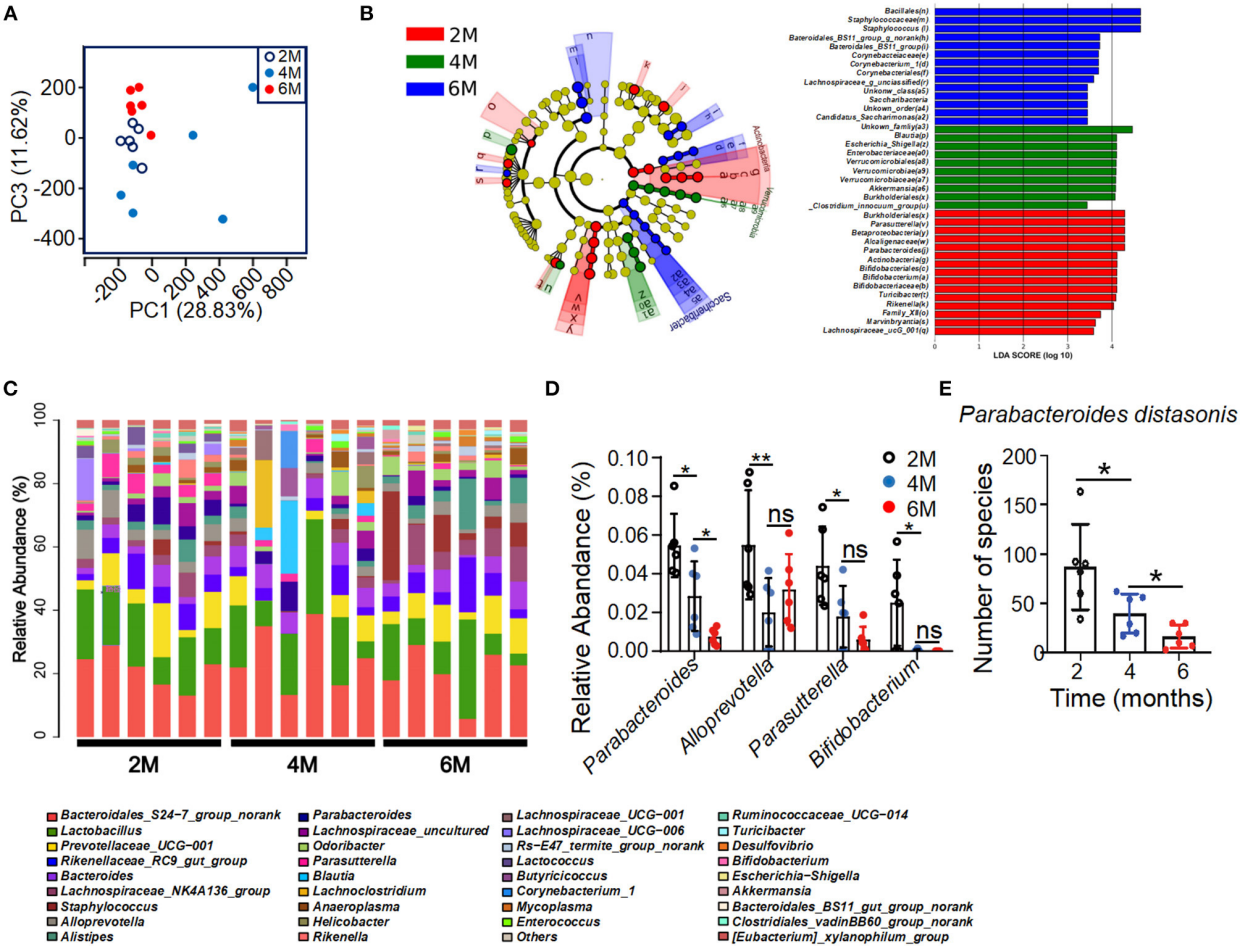
Alteration of bacterial communities in feces of 2-, 4-, and 6-month-old K14-VEGF mice. **(A)** Principal coordinate analysis (PCoA) plot of the fecal microbiota of the 2-, 4-, and 6-month-old K14-VEGF mice (*n* = 6 per group). **(B)** Bacterial 16S rRNA sequences in the 2-, 4-, and 6-month-old mice feces are depicted. The taxonomic cladogram showed the phylogenetic distribution of differentially enriched taxa among three groups. Fecal of 6 month-old-mouse-enriched taxa were presented in blue, fecal of 4-month-old mouse-enriched taxa in green, and fecal of 2-month-old mouse-enriched taxa in red, respectively. Only taxa meeting the criteria (LDA score >4 and *P* < 0.05) were shown. Linear discriminant analysis (LDA) scores revealed bacterial taxa with obvious difference in feces from three groups. Taxa significantly enriched in 2-, 4-, and 6-month-old mice were depicted in red, green, and blue, respectively. **(C)** Gut microbial composition was evaluated in order to determine the relative abundance of genera at three groups. **(D)** Relative abundance of *Parabacteroides, Alloprevotella, Parasutterella*, and *Bifidobacterium* in total fecal microbiota in 2-, 4-, and 6-month-old mice. **(E)** Species number of *Parabacteroides distasonis* in 2-, 4-, and 6-month-old mice. Each symbol represented a mouse, and bars showed means and SEM. Statistical tests used were Student *t*-test in **(E)**. *P < 0.05 and **P < 0.01.

To test whether the metabolic disorder in the K14-VEGF-A-transgenic mice is associated with gut microbiota alterations, 16sRNA gene tag sequencing was performed in order to characterize the gut microbiome in 2-, 4-, and 6-month-old K14-VEGF-A-transgenic mice. 16sRNA gene sequencing demonstrated different microbial communities in the feces of 2-, 4-, and 6-month-old K14-VEGF-A-transgenic mice (Figure 2A). Linear discriminant analysis (LDA) of the gut microbiota of 2-, 4-, and 6-month-old K14-VEGF-A-transgenic mice showed a minimal overlap in the bacterial taxa (Figure 2B). Notably, analysis of prevalent bacterial taxa revealed genus-level differences in the bacterial communities among three groups (Figure 2C). Markable alterations were observed in several taxa, including *Parabacteroides, Parasutterella, Bifidobacterium*, and *Alloprevotella* (Figure 2D). Especially, the relative abundance of *Parabacteroides distasonis* decreased with age (Figure 2E). *P. distasonis* can reduce obesity and related disorders in both ob/ob mice and HFD-fed mice by increasing insulin sensitivity (Wang et al., 2019). Collectively, these data suggest that community-level alterations in the gut microbiota may be associated with the development of psoriasis severity in K14-VEGF-A-transgenic mice. Furthermore, altered profiles of gut microbiota may be associated with metabolic disorders in K14-VEGF-A-transgenic mice.

### Fecal Transfer From 6-Month-Old K14-VEGF-A-Transgenic Mice to 2-Month-Old K14-VEGF-A-Transgenic Mice Deteriorated the Metabolic Disorder

**Figure 3:**
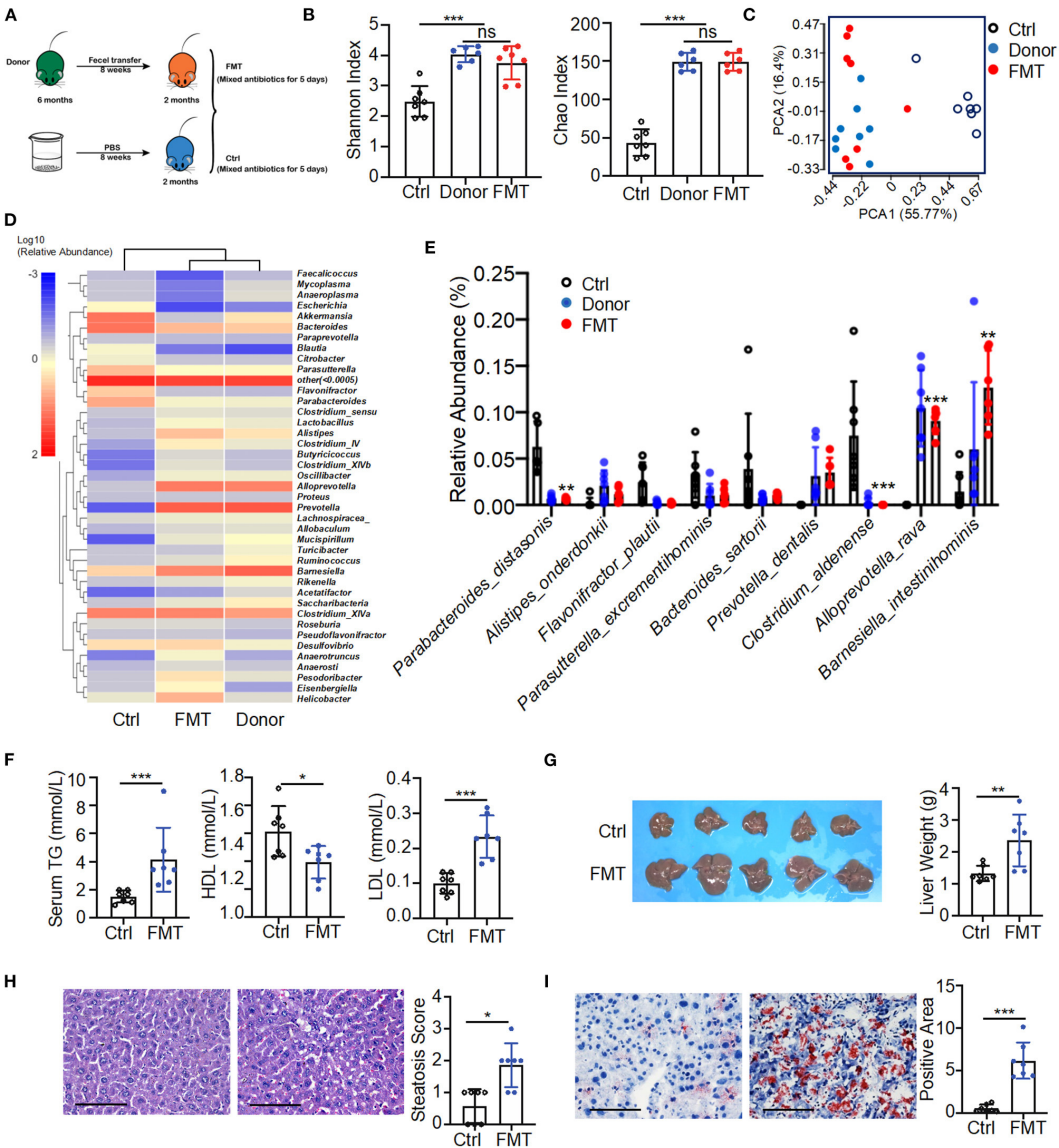
Fecal transfer from 6-month-old K14-VEGF-A-transgenic mice into 2-month-old K14-VEGF-A-transgenic mice exacerbated metabolic disorder. **(A)** Fecal microbiota transplant experimental design. All 2-month-old K14-VEGF mice were treated with mixed antibiotics for 5 days, then transferred with 6-month-old K14-VEGF mice feces as FMT group, or gavaged with sterile PBS as control group for 8 weeks. **(B)** Gut microbiota analysis collected from the control, donor, and FMT groups. Plots of Chao1 richness and Shannon index observed in the FMT, donor, and control groups. **(C)** PCoA plot of the fecal microbiota from the FMT, donor, and control groups (*n* = 7 per group). **(D)** Heat map of differentially expressed fecal metabolites among three groups. Each column represents one group. **(E)** Gut microbial composition was evaluated in order to determine the relative abundance of species at three groups. **(F)** Serum TG, HDL, and LDL from the FMT and control group. **(G)** Liver images and weight of livers. **(H)** Representative HandE-stained sections and degrees of hepatic steatosis. Scale bar, 100 μm. **(I)** Oil Red staining of liver and quantification of hepatic ORO positive area. Scale bar, 100 μm. Each symbol represented a mouse, and bars showed means and SEM. Statistical tests used were Student *t*-test in **(B,E–I)**. *P < 0.05, **P < 0.01, and ***P < 0.001.

To determine the pathogenic potential of gut microbial dysbiosis, fecal transfer from 6-month-old K14-VEGF-A-transgenic mice into 2-month-old K14-VEGF-A-transgenic mice was performed. All 2-month-old mice were treated with mixed antibiotics for 5 days, then were gavaged with feces of 6-month-old mice as fecal microbiota transplantation (FMT) (FMT group) and sterile PBS as a control group for 8 weeks (Figure 3A). Fecal transfer from 6-month-old mice (donor group) exacerbated psoriasis in the FMT group compared with the control group. H&E staining revealed typical histopathological features of psoriasis-like dermatitis, including parakeratosis, Munro's microabscesses, obvious acanthosis with extended rete ridges, and strong inflammatory cell infiltration (Supplementary Figures 4A,B). These data suggest that fecal transfer from 6-month-old K14-VEGF-A-transgenic mice to 2-month-old K14-VEGF-A-transgenic mice deteriorates psoriasis lesion.

The fecal transfer largely affected the gut microbiota distribution of the FMT group. The composition of gut microbiota of the FMT group was significantly changed compared with the control group. The number of taxonomic units in the FMT group was higher than that of the control group, reflecting more alpha diversity (Shannon index, Chao index) in the FMT group (Figure 3B). The FMT and control groups were also distinguished by beta diversity. The FMT group clustered similarly to the donor group (Figure 3C). A heat map of the bacterial composition of the donor, FMT, and control groups showed that the fecal transfer had a significant impact on bacterial composition in the FMT group (Figure 3D). It was further corroborated by abundance differences between the FMT and control groups in species of the gut microbiome, such as a relative reduction in *P. distasonis* and *Clostridium aldenense*, and enrichment in *Alloprevotella rava* and *Barnesiella intestinihominis* in the FMT group (Figure 3E). These findings collectively suggested a strong impact of fecal transfer shift on gut microbial remodeling, notably represented by the abundance decrease in *P. distasonis*. Consistently, a previous study demonstrated the relative abundance of *P. distasonis* significantly decreased in K14-VEGF-A-transgenic mice with age (Figure 2E).

Moreover, the donor group fecal transfer induced elevated serum TG as well as LDL level in the FMT group and also decreased serum HDL level (Figure 3F). Meanwhile, the FMT group exhibited hepatomegaly and increased liver weight compared to the control group, suggesting the increased character of NAFLD (Figure 3G). The donor group fecal transfer induced more liver cell hypertrophy and lipid droplet accumulation in the FMT group than in the control group (Figures 3H,I). These results demonstrate microbiota from the donor group promoted the exacerbation of psoriasis and subsequent metabolic disorder in the FMT group compared to the control group. Overall, fecal transfer from 6-month-old K14-VEGF-A-transgenic mice to 2-month-old K14-VEGF-A-transgenic mice deteriorated metabolic disorder.

We supposed whether fecal transfer from K14-VEGF-A-transgenic mice into FVB mice caused metabolic disorders. FVB mice at 2 months were treated with mixed antibiotics for 5 days, then gavaged with the feces of 6-month-old K14-VEGF-A-transgenic mice as the FMT–FVB group or sterile PBS as FVB control group for 8 weeks (Supplementary Figure 5A). However, no metabolic disorders were observed in FVB mice. There were no differences in body weight and fasting blood glucose (Supplementary Figures 5B,C). To a certain extent, these results suggest that the inflammatory conditions in psoriasis mice contributed to microbiota transplantation from 6-month-old K14-VEGF-A-transgenic mice into 2-month-old K14-VEGF-A-transgenic mice, thereby resulting in metabolic disorders.

### Altered Fecal Metabolic Features in 2-, 4-, and 6-Month-Old K14-VEGF-A-Transgenic Mice

**Figure 4:**
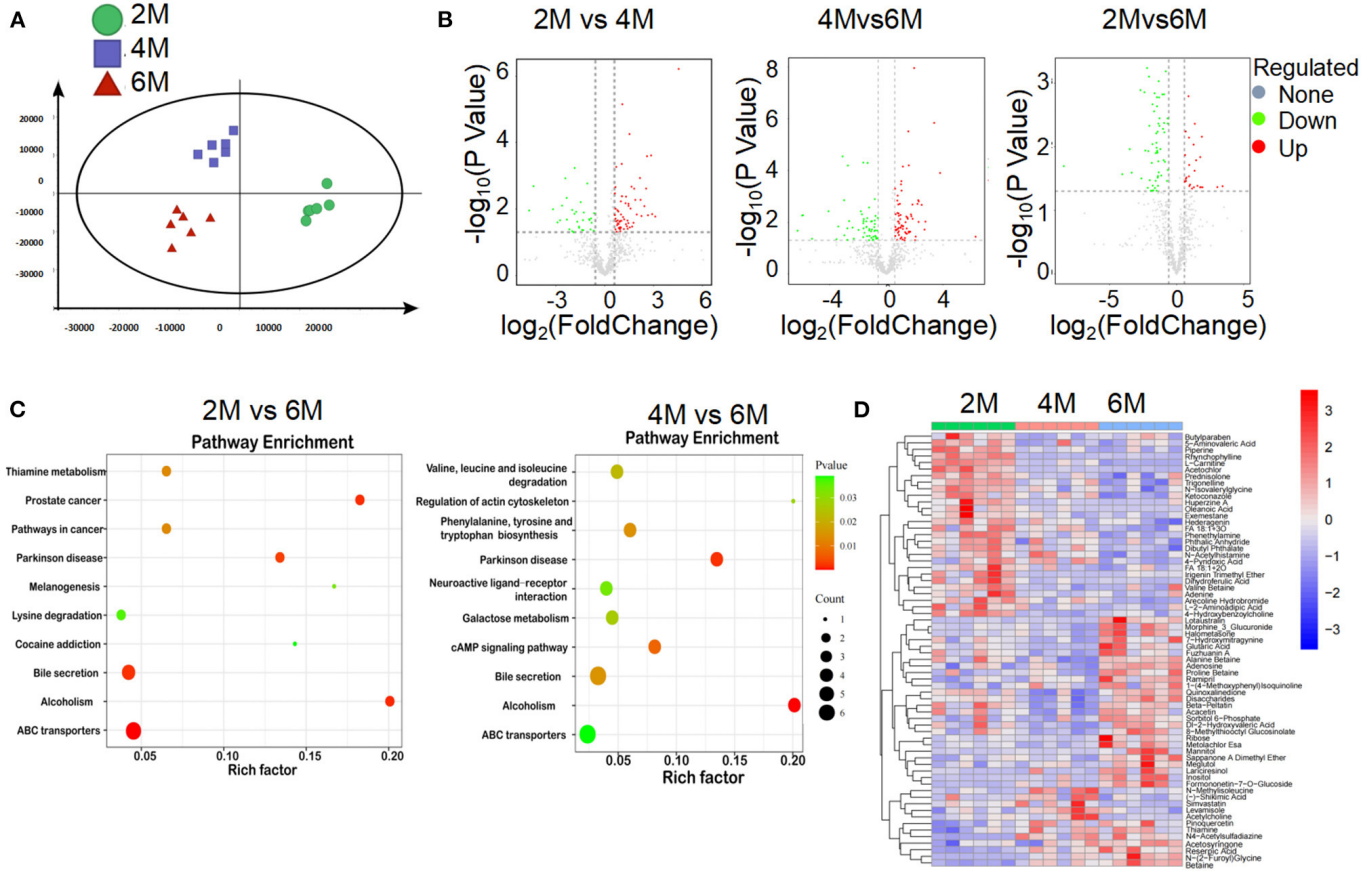
Alteration of gut microbial-associated metabolites in feces of 2-, 4-, and 6-month-old K14-VEGF mice. **(A)** Partial least squares discriminant analysis (PLS-DA) of the fecal metabolites from 2-, 4-, and 6-month-old K14-VEGF mice (*n* = 6 per group). **(B)** Volcano plots of differential gene expression: 2 vs. 4 months, 4 vs. 6 months, and 2 vs. 6 months. **(C)** Enriched KEGG pathways in fecal metabolites of 2 vs. 6 months and 4 vs. 6 months were shown with the *P*-values and the number of metabolites represented in each pathway. The size of each bubble represented the number of metabolites differentially expressed in each pathway, with a scale on the lower right. **(D)** Heat map of differentially expressed fecal metabolites among 2-, 4-, and 6-month-old K14-VEGF mice. Each column represented one mouse. The 2-, 4-, and 6-month-old K14-VEGF mice were depicted in green, red, and blue, respectively.

Many bacterial metabolic end products of the gut play crucial roles in the host metabolic homeostasis and immunological processes, underlining the fundamental importance of these metabolites in human health and disease (Nicholson et al., 2012; Brial et al., 2018). To further clarify the pathological mechanism by which microbiota induce metabolic disorders, we performed an unbiased metabolic screen of the feces of 2, 4, and 6-month-old K14-VEGF-A-transgenic mice. A clear separation was found on the fecal metabolites among the three groups (Figures 4A,B). Kyoto Encyclopedia of Genes and Genomes (KEGG) pathways analyses demonstrated that ABC transporters (ABCT), bile secretion, and alcoholism pathway showed dramatical differences among the three groups, which were associated with host metabolic disorder (Figure 4C). As expected, the heat map showed that metabolic profiles significantly varied in K14-VEGF-A-transgenic mice among three groups of 2, 4, and 6 months, suggesting that microbial metabolic alteration was observed in aged K14-VEGF-A-transgenic mice may be correlated with the development of metabolic disorder (Figure 4D). Based on all these considerations, we concluded that the metabolites might serve as a key mediator in the regulation of gut microbiota on host metabolism.

### Fecal Transfer From a Donor Into the FMT Group Changed the Distribution of Bile Acid

**Figure 5:**
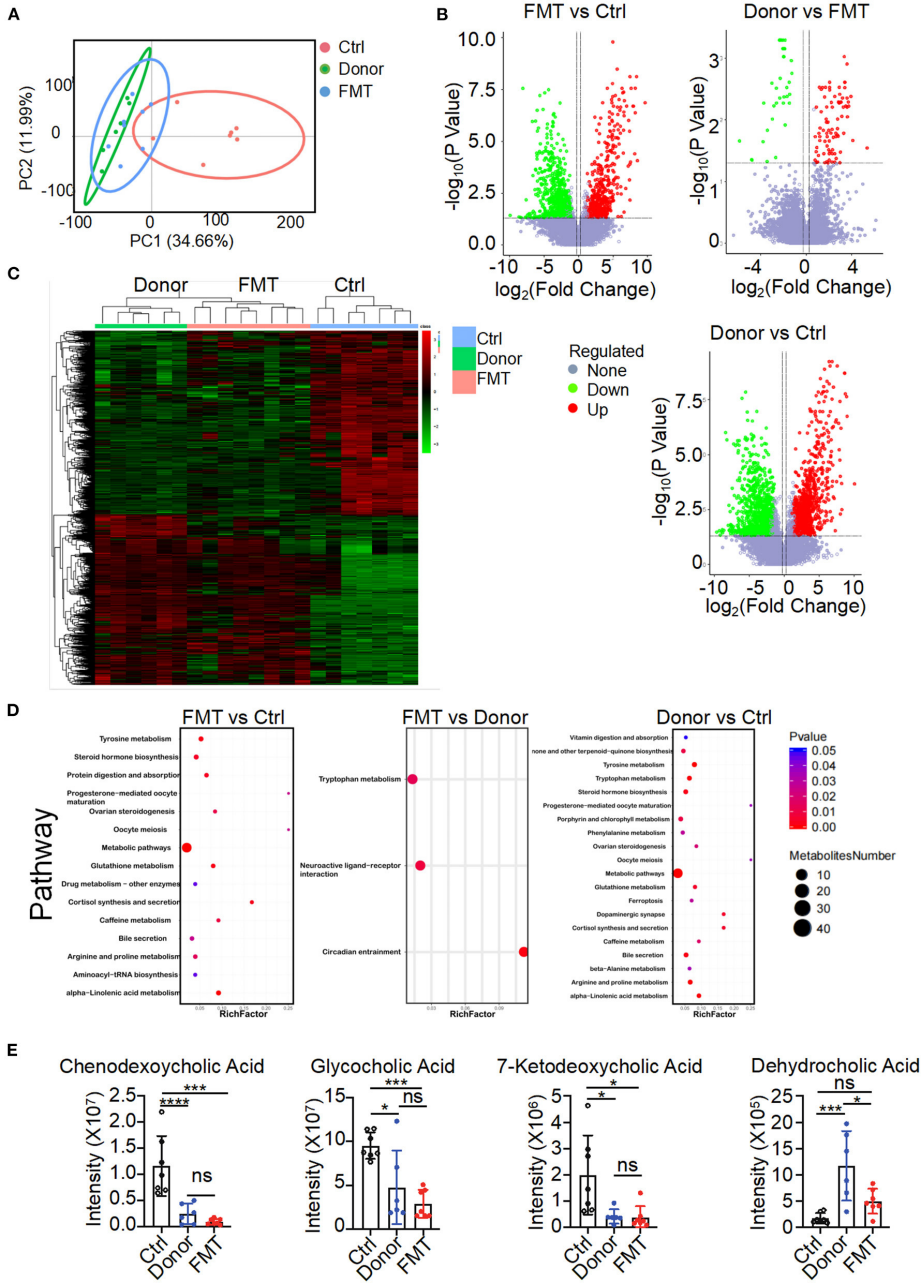
Altered gut microbial-associated metabolite profiles in the FMT group. **(A)** PCoA analysis of fecal metabolomes from the donor, FMT, and control groups (*n* = 6–7). **(B)** Volcano plots of differential gene expression: FMT vs. control, donor vs. FMT, donor vs. control groups. **(C)** Heat map of fecal metabolites composition in the donor, FMT, and control groups. Each column represented a single mouse. Red, overrepresented bacterial genes; green, underrepresented bacterial genes. **(D)** Enriched KEGG pathways in fecal metabolites of FMT vs. control, FMT vs. donor, donor vs. control were shown with the *P*-values and the number of metabolites represented in each pathway. The size of each bubble represented the number of metabolites differentially expressed for each pathway, with a scale on the right (set size) (*n* = 6–7). **(E)** Intensity of chendeoxycholic acid, 7-ketodeoxycholic acid, dehydrocholic acid, and glycocholic acid in feces of the donor, FMT, and control groups is shown (*n* = 6–7). Statistical analysis for **(E)**: Statistical tests used were Student *t*-test. Each symbol represents a mouse; bars represent means and SEM. *P < 0.05, ***P < 0.001, and ****P < 0.0001.

To elucidate the metabolic mechanisms through which gut microbiota colonized in K14-VEGF-A-transgenic mice and induced metabolism disorders, we used untargeted LC/MS to examine the altered metabolites on feces of the donor, FMT, and control groups. When beta diversity was analyzed using the principal component analysis (PCA), we observed clear segregation of fecal metabolite signatures between the FMT and control groups (Figure 5A). FMT and control groups also had different metabolite numbers (Figure 5B). Heatmap of expressed fecal metabolites showed that the donor group fecal transfer induced a significant metabolite composition difference in the FMT group compared with the control group. The FMT and control group fecal metabolites have a distinct fecal metabolome (Figure 5C). Pathway enrichment analysis revealed that the bile secretion pathway varied between FMT and control groups, consistent with the results observed in 2, 4, and 6-month-old K14-VEGF-A-transgenic mice (Figure 5D). Hence, we focused on the bile secretion pathway. It was previously reported that the bile secretion pathway contributed to lipid metabolism. We observed a lower abundance of CDCA, 7-ketodeoxycholic acid, glycocholic acid, and dehydrocholic acid in the FMT group fecal samples compared to the control group (Figure 5E). These data suggest that bile acid metabolized by the gut microbiota is different between the FMT and control groups. Fecal transfer experiments demonstrated that K14-VEGF-A-transgenic mice have an altered metabolite derived from gut microbiota may contribute to the metabolic disease.

### Supplement With CDCA Improved the Metabolic Disorder of K14-VEGF-A-Transgenic Mice

**Figure 6:**
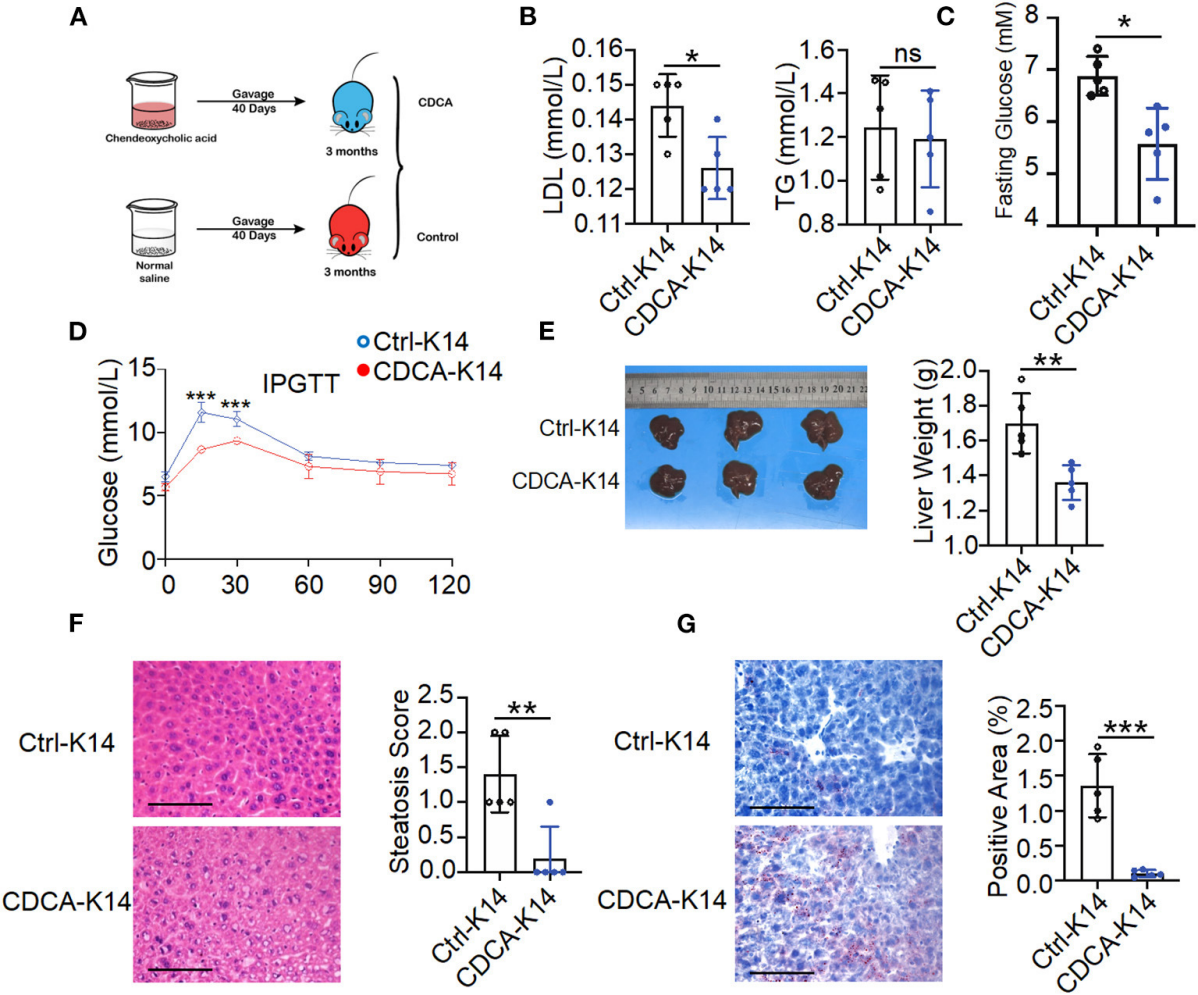
Supplement with CDCA improved metabolic status of K14-VEGF mice. **(A)** Experimental design diagram. Three-month-old K14-VEGF mice (*n* = 5) were gavaged with CDCA (10 mg/kg) as the CDCA-K14 group. Another group was gavaged with solvent as K14-control group. All mice were gavaged for 40 days. **(B)** Serum LDL and TG level. **(C)** Fasting blood glucose levels. **(D)** Blood glucose concentrations following intraperitoneal glucose tolerance tests. **(E)** Representative pictures and weight of livers. **(F)** Representative HandE-stained sections of liver and degrees of hepatic steatosis. Scale bar, 100 μm. **(G)** Representative images of liver tissues Oil Red staining and quantification of hepatic ORO. Scale bar, 100 μm. Statistical analysis for **(A–G)**: Each symbol represents a mouse, and bars represent means and SEM. Statistical tests used were Student's *t*-test in **(B–G)**. *P < 0.05, **P < 0.01, and ***P < 0.001.

Gut microbiota composition alteration might contribute to disturbed microbiota-derived metabolites that influence the host metabolism. CDCA was identified as effective ligands for fafrnesoid X receptor (FXR) (de Aguiar Vallim et al., 2013; de Boer et al., 2018; Jia et al., 2018) and mediated the host lipid metabolism. It can prevent high-fat diet-induced obesity and improve glucose tolerance (Chen et al., 2017). To evaluate the effects of CDCA on metabolic disorders in K14-VEGF-A-transgenic mice, we treated 3-month-old K14-VEGF-A-transgenic mice with vehicle or CDCA by oral gavage daily for a period of 40 days (Figure 6A). Compared to the vehicle-treated K14-VEGF-A-transgenic group, CDCA effectively improved blood dyslipidemia in K14-VEGF-A-transgenic mice by reducing the concentrations of serum LDL (Figure 6B), and the CDCA-treated group showed a significant improvement in the fasting blood glucose (Figure 6C). Moreover, glucose tolerance significantly ameliorated in K14-VEGF-A-transgenic mice compared with that in the FVB group (Figure 6D). We found that CDCA greatly improved the liver weight of K14-VEGF-A-transgenic mice (Figure 6E). CDCA treatment apparently reduced macrosteatosis, hepatocyte ballooning, and lipid deposition in the livers of K14-VEGF-A-transgenic mice, as indicated by H&E and Oil Red O staining of liver sections (Figures 6F,G). Overall, CDCA treatment effectively reversed metabolic disorders in K14-VEGF-A-transgenic mice, which is in line with a previous study (Chen et al., 2017).

## Discussion

This study investigated the role of gut microbiota in metabolic dysfunction and its underlying mechanism in a psoriasis mice model by fecal transfer. Several studies have reported that patients with psoriasis had an increased chance of metabolic disorders, a combination of disorders, such as obesity, diabetes mellitus, and dyslipidemia (Khalid et al., 2013; Candia et al., 2015; Lonnberg et al., 2016; Rutter et al., 2016; Rodriguez-Zuniga and Garcia-Perdomo, 2017; Takeshita et al., 2017). However, an integrated understanding of the high prevalence of metabolic disorders in patients with psoriasis is still elusive. Here, by using genetic-induced animal models of psoriasis, we demonstrated that altered microbiota are critical for the maintenance of metabolic homeostasis in K14-VEGF-A-transgenic mice. To the best of our knowledge, our study originally researched the pathological mechanism of metabolic dysfunction in psoriasis and the novel role of gut microbiota-associated bile acid in regulating metabolic dysfunction in psoriasis mice.

Frist, we demonstrated that gut microbiota dysbiosis may critically associate with the metabolic disorders in the psoriasis mice. By comparing FVB mice at the age of 2, 4, and 6 months, we observed that the K14-VEGF-A-transgenic mouse model aggravated psoriasis-like dermatitis, which is consistent with the previous study (Xia et al., 2003). Meanwhile, we found that metabolic disorder is also exacerbated in K14-VEGF-A-transgenic mice with increasing age. Previous studies have confirmed that the gut microbiota composition of patients with psoriasis was different from that of the healthy subjects (Codoner et al., 2018; Hidalgo-Cantabrana et al., 2019; Dei-Cas et al., 2020). We found K14-VEGF-A-transgenic mice have different microbial communities at the age of 2, 4, and 6 months. Hence, differential bacterial communities in the K14-VEGF-A-transgenic mouse model were possibly associated with psoriasis severity with increased age. In this study, we observed that the abundance of several microbial taxa, including *Parabacteroides, Parasutterella, Bifidobacterium*, and *Alloprevotella* alerted with age, suggesting the increased age had a pronounced effect on the gut microbiota composition of the K14-VEGF-A-transgenic mice. Among them, *Bifidobacterium* (Da Silva et al., 2020) and *Parabacteroides* (Wu et al., 2019) had abilities to improve body metabolism and promote host health. Thus, *Bifidobacterium* and *Parabacteroides* may be negatively correlated with the severity of metabolic abnormalities. In species levels, the relative abundance of *P. distasonis* decreased in K14-VEGF-A-transgenic mice as the lesions of psoriasis worsened. A study revealed that *P. distasonis* significantly decreased in patients with psoriasis compared with healthy individuals (Shapiro et al., 2019). Interestingly, the abundance of *P. distasonis* is relatively lower in patients with obesity and NAFLD (Verdam et al., 2013; Del Chierico et al., 2017). *P. distasonis was* a predominant bacterial group in the hosts that can maintain important physiological functions. A previous study has demonstrated oral treatment with live *P. distasonis* reduced weight gain, improved glucose homeostasis, and corrected obesity-related abnormalities, including hyperlipidemia and hepatic steatosis, in both ob/ob mice and high-fat diet-induced metabolic syndrome mice (Wang et al., 2019). Based on these studies, we assumed that the metabolic dysfunction of K14-VEGF-A-transgenic mice may be related to gut microbes.

The microbiota composition and function alternations caused by factors such as genetics and lifestyle have been identified as critical contributors to the worldwide epidemics of metabolic diseases (Ridaura et al., 2013). We found that transmission of the fecal microbiota from 6-month-old K14-VEGF-A-transgenic into 2-month-old K14-VEGF-A-transgenic mice accelerated the metabolic disorders. Fecal microbiota transfer can induce hyperlipidemia and fatty liver, which are phenotypes implicated in metabolic disease. Quantitative microbiome alterations were associated with metabolic disorders. The reduction in bacterial species with potential protection on the host can result in metabolic dysfunction (Goodman and Gordon, 2010). We found that *P. distasonis*, which promotes lipid metabolism and exerts a potentially protective effect on the host, was decreased in the FMT group. Thus, these results suggested that gut microbiota alterations in the K14-VEGF-A-transgenic mouse are involved in metabolic homeostasis. However, fecal microbiota transfer from 6-month-old K14-VEGF-A-transgenic mice into 2-month-old FVB mice induces no metabolic-associated disorder. It can be explained that the gut microbiota of K14-VEGF-A-transgenic mice probably had no ability to colonize in FVB and trigger the metabolic disorder. Notably, compared with FVB mice, K14-VEGF-A-transgenic mice were at a systemic inflammatory status with increased release of pro-inflammatory cytokines from immune-related cells and chronic activation of immune systems (Shibuya, 2009; Suzuki et al., 2014; Liang et al., 2017). Severe inflammatory activation affects the structure and composition of the microbiota (Belkaid and Harrison, 2017), suppressing the trigger of metabolic disorder in FVB mice. Thus, we hypothesized that in K14-VEGF-A-transgenic mice, psoriasis susceptibility genes triggered autoimmune activation, potentially promoting gut microbiota dysbiosis, and then inducing metabolic disorder.

Microbiome alterations can systemically impact distant disease progression *via* the release of metabolites (Postler and Ghosh, 2017). Specific bacterial metabolites, such as the short-chain fatty acids (SCFAs) produced from the fermentation of non-digestible carbohydrates, effect host metabolism (Canfora et al., 2015). However, gut microbiota-derived metabolite signature in psoriasis has not been investigated. Here, we elucidated metabolites induced by gut microbiota alteration component altered in 2-, 4-, and 6-month-old K14-VEGF-A-transgenic mice. Metabolome analysis detected several pathways including bile secretion, alcoholism, and ABCTs changed. Among them, ABCTs are well-known to control the metabolism and transport lipid particles. ABCT directly engaged in the production, storage, and transport of cholesterol to maintain cholesterol equilibrium (Tarling et al., 2013). Thus, alterations in ABCTs contribute to the development of metabolic disorders (Ye et al., 2020; Behl et al., 2021). Bile acids secreted by bile were appreciated as metabolic integrators and signaling factors. Bile acid can control glucose, lipid, and energy metabolism by binding to the nuclear hormone FXR and Takeda G protein receptor 5 (TGR5) in multiple organs (Shapiro et al., 2018). It has been reported that *P. distasonis* have the capacity to transform bile acids and dramatically altered the bile acid profile (Wang et al., 2019). A number of bile-acid-activated signaling pathways were selected as crucial therapeutic targets for metabolic disorders (Thomas et al., 2008). Hence, in K14-VEGF-A-transgenic mice, metabolite changes induced by gut microbiota were essential for regulating the lipid metabolism *via* specific metabolic pathways.

Consistently, there was a significant difference in fecal metabolites of the FMT and control groups. In the FMT group, the bile secretion pathway and bile acid profile were significantly changed. The CDCA decreased in fecal samples of the FMT group. CDCA is one of the primary bile acids synthesized in the human liver (Iser and Sali, 1981). A previous study revealed the CDCA supplement can effectively increase brown adipose tissue activities and energy expenditure, which is an effective method for counteracting obesity and related metabolic diseases (Broeders et al., 2015). After patients with hypertriglyceridaemia were treated with CDCA, serum TG level decreased (Bateson et al., 1978). CDCA increased glucagon-like peptide-1 and glucagon secretion (Hansen et al., 2016). It can improve insulin resistance by inhibiting proinflammatory adipokines and enhancing major anti-inflammatory and insulin-sensitizing adipokines (Shihabudeen et al., 2015). As an endogenous FXR ligand (de Aguiar Vallim et al., 2013), CDCA induced the expression of endocrine hormone fibroblast growth factor 19 (FGF19) in humans (Song et al., 2009). FGF19 suppressed hepatic lipogenesis and increases hepatic fatty acid oxidation, thereby reducing body weight gain from diet-induced obesity (Fang et al., 2015). In this study, we showed that the CDCA supplement by gavage exerted a protective effect on the metabolic disorder in K14-VEGF-A-transgenic mice, consistent with a previous study (Chen et al., 2017). Overall, these results suggested a functional link between gut microbial dysbiosis, gut metabolites, and metabolic disorder in psoriasis mice.

There are several limitations to this study. Despite we have clarified gut microbial dysbiosis and altered metabolism pathways in the psoriasis-prone K14-VEGF-A-transgenic mice, (i) mechanistically, we did not identify the bacterial species responsible for bile acid metabolite alteration that may contribute to metabolic disorders in the FMT group; (ii) we did not detect whether oral CDCA supplement significantly increased CDCA levels in K14-VEGF-A-transgenic mice; (iii) moreover, although we have documented metabolic disorders in FMT mice in response to gut microbiota variations, the elaborate molecular mechanism was warranted in the further research.

In summary, compared to aged-matched FVB, the metabolic disorder risk increased in K14-VEGF-A-transgenic mice with age. Meanwhile, gut microbiota communities changed in the feces of 2-, 4-, and 6-month-old K14-VEGF-A-transgenic mice. Fecal transfer from 6-month-old K14-VEGF-A-transgenic mice to 2-month-old K14-VEGF-A-transgenic mice deteriorated the metabolic disorder in recipient mice. Moreover, fecal metabolites altered in 2-, 4-, and 6-month-old K14-VEGF-A-transgenic mice. After fecal transfer from 6-month-old mice into 2-month-old mice, bile acid distribution changed in recipient mice. Reversely, the CDCA supplement ameliorated the metabolic disorder in K14-VEGF-A-transgenic mice. Gut microbial metabolites may be potential targets to prevent and treat the metabolic disorder in psoriasis.

## Data Availability Statement

The data presented in the study are deposited in the Sequence Read Archive (SRA) repository, BioProject ID: PRJNA804695.

## Ethics Statement

The animal study was reviewed and approved by Institutional Animal Care and Treatment Committee of Sichuan University (Chengdu, PR China).

## Author Contributions

JL: conception and design of the study, editing and revising the article writing, and is the guarantor of this study. YHa and PZ: acquisition of data. Y-jZ and YHa: drafting the manuscript. SZ: analysis and interpretation of data and helping on methodology. PZ: visualization of some metabolomic data. QZ, JY, and YHu: revising the manuscript. All authors read and approved the final manuscript.

## Funding

This work was supported by the National Natural Science Foundation of China [81673061, 81472650, and 31271483], the National Science and Technology Major Project [2018ZX09303006-001-006 and 2019ZX09201004-003], and the Key Research and Development Program of Sichuan Province [2020YFS0271].

## Conflict of Interest

The authors declare that the research was conducted in the absence of any commercial or financial relationships that could be construed as a potential conflict of interest.

## Publisher's Note

All claims expressed in this article are solely those of the authors and do not necessarily represent those of their affiliated organizations, or those of the publisher, the editors and the reviewers. Any product that may be evaluated in this article, or claim that may be made by its manufacturer, is not guaranteed or endorsed by the publisher.

## References

[B1] Baker B. S. Brent L. Valdimarsson H. Powles A. V. al-Imara L. Walker M.. (1992). Is epidermal cell proliferation in psoriatic skin grafts on nude mice driven by T-cell derived cytokines? Br. J. Dermatol. 126, 105–110. 10.1111/j.1365-2133.1992.tb07805.x 1536776

[B2] Bateson M. C. Maclean D. Evans J. R. Bouchier I. A. (1978). Chenodeoxycholic acid therapy for hypertriglyceridaemia in men. Br. J. Clin. Pharmacol. 5, 249–254. 10.1111/j.1365-2125.1978.tb01632.x 656270 PMC1429265

[B3] Behl T. Sehgal A. Grover M. Singh S. Sharma N. Bhatia S. (2021). Uncurtaining the pivotal role of ABC transporters in diabetes mellitus. Environ. Sci. Pollut. Res. Int. 28, 41533–41551. 10.1007/s11356-021-14675-y 34085197

[B4] Belkaid Y. Harrison O. J. (2017). Homeostatic immunity and the microbiota. Immunity 46, 562–576. 10.1016/j.immuni.2017.04.008 28423337 PMC5604871

[B5] Boehncke W. H. Schon M. P. (2015). Psoriasis. Lancet 386, 983–994. 10.1016/S0140-6736(14)61909-7 26025581

[B6] Brial F. Le Lay A. Dumas M. E. Gauguier D. (2018). Implication of gut microbiota metabolites in cardiovascular and metabolic diseases. Cell. Mol. Life Sci. 75, 3977–3990. 10.1007/s00018-018-2901-1 30101405 PMC6182343

[B7] Broeders E. P. Nascimento E. B. Havekes B. Brans B. Roumans K. H. Tailleux A. (2015). The bile acid chenodeoxycholic acid increases human brown adipose tissue activity. Cell Metab. 22, 418–426. 10.1016/j.cmet.2015.07.002 26235421

[B8] Candia R. Ruiz A. Torres-Robles R. Chavez-Tapia N. Mendez-Sanchez N. Arrese M. (2015). Risk of non-alcoholic fatty liver disease in patients with psoriasis: a systematic review and meta-analysis. J. Eur. Acad. Dermatol. Venereol. 29, 656–662. 10.1111/jdv.12847 25418531

[B9] Canfora E. E. Jocken J. W. Blaak E. E. (2015). Short-chain fatty acids in control of body weight and insulin sensitivity. Nat. Rev. Endocrinol. 11, 577–591. 10.1038/nrendo.2015.128 26260141

[B10] Canfora E. E. Meex R. C. R. Venema K. Blaak E. E. (2019). Gut microbial metabolites in obesity, NAFLD and T2DM. Nat. Rev. Endocrinol. 15, 261–273. 10.1038/s41574-019-0156-z 30670819

[B11] Cani P. D. (2019). Microbiota and metabolites in metabolic diseases. Nat. Rev. Endocrinol. 15, 69–70. 10.1038/s41574-018-0143-9 30602737

[B12] Chen X. Yan L. Guo Z. Chen Y. Li M. Huang C.. (2017). Chenodeoxycholic acid attenuates high-fat diet-induced obesity and hyperglycemia via the G protein-coupled bile acid receptor 1 and proliferator-activated receptor gamma pathway. Exp. Ther. Med. 14, 5305–5312. 10.3892/etm.2017.5232 29285057 PMC5740767

[B13] Cheng J. Palva A. M. de Vos W. M. Satokari R. (2013). Contribution of the intestinal microbiota to human health: from birth to 100 years of age. Curr. Top. Microbiol. Immunol. 358, 323–346. 10.1007/82_2011_189 22094893

[B14] Codoner F. M. Ramirez-Bosca A. Climent E. Carrion-Gutierrez M. Guerrero M. Perez-Orquin J. M. (2018). Gut microbial composition in patients with psoriasis. Sci. Rep. 8, 3812. 10.1038/s41598-018-22125-y 29491401 PMC5830498

[B15] Da Silva C. C. Monteil M. A. Davis E. M. (2020). Overweight and obesity in children are associated with an abundance of firmicutes and reduction of bifidobacterium in their gastrointestinal microbiota. Child. Obes. 16, 204–210. 10.1089/chi.2019.0280 31934770

[B16] de Aguiar Vallim T. Q. Tarling E. J. Edwards P. A. (2013). Pleiotropic roles of bile acids in metabolism. Cell Metab. 17, 657–669. 10.1016/j.cmet.2013.03.013 23602448 PMC3654004

[B17] de Boer J. F. Bloks V. W. Verkade E. Heiner-Fokkema M. R. Kuipers F. (2018). New insights in the multiple roles of bile acids and their signaling pathways in metabolic control. Curr. Opin. Lipidol. 29, 194–202. 10.1097/MOL.0000000000000508 29553998

[B18] Dei-Cas I. Giliberto F. Luce L. Dopazo H. Penas-Steinhardt A. (2020). Metagenomic analysis of gut microbiota in non-treated plaque psoriasis patients stratified by disease severity: development of a new Psoriasis-Microbiome Index. Sci. Rep. 10, 12754. 10.1038/s41598-020-69537-3 32728075 PMC7391695

[B19] Del Chierico F. Nobili V. Vernocchi P. Russo A. De Stefanis C. Gnani D.. (2017). Gut microbiota profiling of pediatric nonalcoholic fatty liver disease and obese patients unveiled by an integrated meta-omics-based approach. Hepatology 65, 451–464. 10.1002/hep.28572 27028797

[B20] Fan Y. Pedersen O. (2021). Gut microbiota in human metabolic health and disease. Nat Rev Microbiol 19, 55–71. 10.1038/s41579-020-0433-9 32887946

[B21] Fang S. Suh J. M. Reilly S. M. Yu E. Osborn O. Lackey D.. (2015). Intestinal FXR agonism promotes adipose tissue browning and reduces obesity and insulin resistance. Nat. Med. 21, 159–165. 10.1038/nm.3760 25559344 PMC4320010

[B22] Goodman A. L. Gordon J. I. (2010). Our unindicted coconspirators: human metabolism from a microbial perspective. Cell Metab. 12, 111–116. 10.1016/j.cmet.2010.07.001 20674856 PMC2935662

[B23] Gui X. Y. Yu X. L. Jin H. Z. Zuo Y. G. Wu C. (2018). Prevalence of metabolic syndrome in Chinese psoriasis patients: a hospital-based cross-sectional study. J. Diabetes Investig. 9, 39–43. 10.1111/jdi.12663 28371532 PMC5754527

[B24] Hansen M. Scheltema M. J. Sonne D. P. Hansen J. S. Sperling M. Rehfeld J. F. (2016). Effect of chenodeoxycholic acid and the bile acid sequestrant colesevelam on glucagon-like peptide-1 secretion. Diabetes Obes. Metab. 18, 571–580. 10.1111/dom.12648 26888164

[B25] Hidalgo-Cantabrana C. Gomez J. Delgado S. Requena-Lopez S. Queiro-Silva R. Margolles A. (2019). Gut microbiota dysbiosis in a cohort of patients with psoriasis. Br. J. Dermatol. 181, 1287–1295. 10.1111/bjd.17931 30920647

[B26] Holmannova D. Borsky P. Borska L. Andrys C. Hamakova K. Rehacek V. (2020). Metabolic syndrome, clusterin and elafin in patients with Psoriasis Vulgaris. Int. J. Mol. Sci. 21, 5617. 10.3390/ijms21165617 32764517 PMC7460615

[B27] Iser J. H. Sali A. (1981). Chenodeoxycholic acid: a review of its pharmacological properties and therapeutic use. Drugs 21, 90–119. 10.2165/00003495-198121020-00002 7009140

[B28] Jia W. Xie G. Jia W. (2018). Bile acid-microbiota crosstalk in gastrointestinal inflammation and carcinogenesis. Nat. Rev. Gastroenterol. Hepatol. 15, 111–128. 10.1038/nrgastro.2017.119 29018272 PMC5899973

[B29] Khalid U. Hansen P. R. Gislason G. H. Lindhardsen J. Kristensen S. L. Winther S. A. (2013). Psoriasis and new-onset diabetes: a Danish nationwide cohort study. Diabetes Care 36, 2402–2407. 10.2337/dc12-2330 23491525 PMC3714512

[B30] Koeth R. A. Wang Z. Levison B. S. Buffa J. A. Org E. Sheehy B. T. (2013). Intestinal microbiota metabolism of L-carnitine, a nutrient in red meat, promotes atherosclerosis. Nat. Med. 19, 576–585. 10.1038/nm.3145 23563705 PMC3650111

[B31] Le Chatelier E. Nielsen T. Qin J. Prifti E. Hildebrand F. Falony G. (2013). Richness of human gut microbiome correlates with metabolic markers. Nature 500, 541–546. 10.1038/nature12506 23985870

[B32] Lebwohl M. (2003). Psoriasis. Lancet 361, 1197–1204. 10.1016/S0140-6736(03)12954-6 12686053

[B33] Liang Y. Sarkar M. K. Tsoi L. C. Gudjonsson J. E. (2017). Psoriasis: a mixed autoimmune and autoinflammatory disease. Curr. Opin. Immunol. 49, 1–8. 10.1016/j.coi.2017.07.007 28738209 PMC5705427

[B34] Lonnberg A. S. Skov L. Skytthe A. Kyvik K. O. Pedersen O. B. Thomsen S. F. (2016). Association of Psoriasis with the risk for type 2 diabetes mellitus and obesity. JAMA Dermatol. 152, 761–767. 10.1001/jamadermatol.2015.6262 27120802

[B35] Malik S. Wong N. D. Franklin S. S. Kamath T. V. L'Italien G. J. Pio J. R.. (2004). Impact of the metabolic syndrome on mortality from coronary heart disease, cardiovascular disease, and all causes in United States adults. Circulation 110, 1245–1250. 10.1161/01.CIR.0000140677.20606.0E 15326067

[B36] Manolis A. A. Manolis T. A. Melita H. Manolis A. S. (2019). Psoriasis and cardiovascular disease: the elusive link. Int. Rev. Immunol. 38, 33–54. 10.1080/08830185.2018.1539084 30457023

[B37] Mottillo S. Filion K. B. Genest J. Joseph L. Pilote L. Poirier P. (2010). The metabolic syndrome and cardiovascular risk a systematic review and meta-analysis. J. Am. Coll. Cardiol. 56, 1113–1132. 10.1016/j.jacc.2010.05.034 20863953

[B38] Nicholson J. K. Holmes E. Kinross J. Burcelin R. Gibson G. Jia W. (2012). Host-gut microbiota metabolic interactions. Science 336, 1262–1267. 10.1126/science.1223813 22674330

[B39] Postler T. S. Ghosh S. (2017). Understanding the holobiont: how microbial metabolites affect human health and shape the immune system. Cell Metab. 26, 110–130. 10.1016/j.cmet.2017.05.008 28625867 PMC5535818

[B40] Ren X. Li J. Zhou X. Luo X. Huang N. Wang Y.. (2009). Recombinant murine interleukin 4 protein therapy for psoriasis in a transgenic VEGF mouse model. Dermatology 219, 232–238. 10.1159/000235974 19729876

[B41] Ridaura V. K. Faith J. J. Rey F. E. Cheng J. Duncan A. E. Kau A. L. (2013). Gut microbiota from twins discordant for obesity modulate metabolism in mice. Science 341, 1241214. 10.1126/science.1241214 24009397 PMC3829625

[B42] Rodriguez-Zuniga M. J. M. Garcia-Perdomo H. A. (2017). Systematic review and meta-analysis of the association between psoriasis and metabolic syndrome. J. Am. Acad. Dermatol. 77, 657–666.e658. 10.1016/j.jaad.2017.04.1133 28917453

[B43] Rutter M. K. Kane K. Lunt M. Cordingley L. Littlewood A. Young H. S. (2016). Primary care-based screening for cardiovascular risk factors in patients with psoriasis. Br. J. Dermatol. 175, 348–356. 10.1111/bjd.14557 26990294 PMC5113692

[B44] Shapiro H. Kolodziejczyk A. A. Halstuch D. Elinav E. (2018). Bile acids in glucose metabolism in health and disease. J. Exp. Med. 215, 383–396. 10.1084/jem.20171965 29339445 PMC5789421

[B45] Shapiro J. Cohen N. A. Shalev V. Uzan A. Koren O. Maharshak N. (2019). Psoriatic patients have a distinct structural and functional fecal microbiota compared with controls. J. Dermatol. 46, 595–603. 10.1111/1346-8138.14933 31141234

[B46] Shi Q. Z. Wang L. W. Zhang W. Gong Z. J. (2010). Betaine inhibits toll-like receptor 4 expression in rats with ethanol-induced liver injury. World J. Gastroenterol. 16, 897–903. 10.3748/wjg.v16.i7.897 20143470 PMC2825338

[B47] Shibuya M. (2009). Unique signal transduction of the VEGF family members VEGF-A and VEGF-E. Biochem. Soc. Trans. 37, 1161–1166. 10.1042/BST0371161 19909239

[B48] Shihabudeen M. S. Roy D. James J. Thirumurugan K. (2015). Chenodeoxycholic acid, an endogenous FXR ligand alters adipokines and reverses insulin resistance. Mol. Cell. Endocrinol. 414, 19–28. 10.1016/j.mce.2015.07.012 26188168

[B49] Sommer F. Backhed F. (2013). The gut microbiota–masters of host development and physiology. Nat. Rev. Microbiol. 11, 227–238. 10.1038/nrmicro2974 23435359

[B50] Song K. H. Owsley L. i. T. Strom E. Chiang S. Bile J. Y. (2009). acids activate fibroblast growth factor 19 signaling in human hepatocytes to inhibit cholesterol 7alpha-hydroxylase gene expression. Hepatology 49, 297–305. 10.1002/hep.22627 19085950 PMC2614454

[B51] Suzuki T. Hirakawa S. Shimauchi T. Ito T. Sakabe J. Detmar M. (2014). VEGF-A promotes IL-17A-producing gammadelta T cell accumulation in mouse skin and serves as a chemotactic factor for plasmacytoid dendritic cells. J. Dermatol. Sci. 74, 116–124. 10.1016/j.jdermsci.2013.12.013 24485663

[B52] Takeshita J. Grewal S. Langan S. M. Mehta N. N. Ogdie A. Van Voorhees A. S. (2017). Psoriasis and comorbid diseases: epidemiology. J. Am. Acad. Dermatol. 76, 377–390. 10.1016/j.jaad.2016.07.064 28212759 PMC5731650

[B53] Tarling E. J. Aguiar Vallim d. e. Edwards T. Q. Role P. A. (2013). of ABC transporters in lipid transport and human disease. Trends Endocrinol. Metab. 24, 342–350. 10.1016/j.tem.2013.01.006 23415156 PMC3659191

[B54] Thomas C. Pellicciari R. Pruzanski M. Auwerx J. Schoonjans K. (2008). Targeting bile-acid signalling for metabolic diseases. Nat. Rev. Drug Discov. 7, 678–693. 10.1038/nrd2619 18670431

[B55] van der Fits L. Mourits S. Voerman J. S. Kant M. Boon L. Laman J. D. (2009). Imiquimod-induced psoriasis-like skin inflammation in mice is mediated via the IL-23/IL-17 axis. J. Immunol. 182, 5836–5845. 10.4049/jimmunol.0802999 19380832

[B56] Verdam F. J. Fuentes S. de Jonge C. Zoetendal E. G. Erbil R. Greve J. W.. (2013). Human intestinal microbiota composition is associated with local and systemic inflammation in obesity. Obesity 21, E607–E615. 10.1002/oby.20466 23526699

[B57] Wang K. Liao M. Zhou N. Bao L. Ma K. Zheng Z. (2019). *Parabacteroides distasonis* alleviates obesity and metabolic dysfunctions via production of succinate and secondary bile acids. Cell Rep. 26, 222–235.e225. 10.1016/j.celrep.2018.12.028 30605678

[B58] Wang Z. Klipfell E. Bennett B. J. Koeth R. Levison B. S. Dugar B. (2011). Gut flora metabolism of phosphatidylcholine promotes cardiovascular disease. Nature 472, 57–63. 10.1038/nature09922 21475195 PMC3086762

[B59] Wu H. Tremaroli V. Backhed F. (2015). Linking microbiota to human diseases: a systems biology perspective. Trends Endocrinol. Metab. 26, 758–770. 10.1016/j.tem.2015.09.011 26555600

[B60] Wu T. R. Lin C. S. Chang C. J. Lin T. L. Martel J. Ko Y. F. (2019). Gut commensal *Parabacteroides goldsteinii* plays a predominant role in the anti-obesity effects of polysaccharides isolated from *Hirsutella sinensis*. Gut 68, 248–262. 10.1136/gutjnl-2017-315458 30007918

[B61] Xia Y. P. Hylton L. i. B. Detmar D. Yancopoulos M. Rudge G. D. Transgenic J. S. (2003). delivery of VEGF to mouse skin leads to an inflammatory condition resembling human psoriasis. Blood 102, 161–168. 10.1182/blood-2002-12-3793 12649136

[B62] Ye Z. Lu Y. Wu T. (2020). The impact of ATP-binding cassette transporters on metabolic diseases. Nutr. Metab. 17, 61. 10.1186/s12986-020-00478-4 32774439 PMC7398066

[B63] Zhang X. L. Wang Y. N. Ma L. Y. Liu Z. S. Ye F. Yang J. H. (2020). Uncarboxylated osteocalcin ameliorates hepatic glucose and lipid metabolism in KKAy mice via activating insulin signaling pathway. Acta Pharmacol. Sin. 41, 383–393. 10.1038/s41401-019-0311-z 31659239 PMC7470804

[B64] Zhao L. Zhang F. Ding X. Wu G. Lam Y. Y. Wang X. (2018). Gut bacteria selectively promoted by dietary fibers alleviate type 2 diabetes. Science 359, 1151–1156. 10.1126/science.aao5774 29590046

