# Supplementary material for: Gut Microbiota Dysbiosis and Altered Bile Acid Catabolism Lead to Metabolic Disorder in Psoriasis Mice
Source: Front Microbiol. 2022 Apr 14;13:853566. doi: 10.3389/fmicb.2022.853566 (PMC9048827; doi:10.3389/fmicb.2022.853566)
Supplement: Supplementary file 1 [file Data_Sheet_1.docx]

Supplementary Material


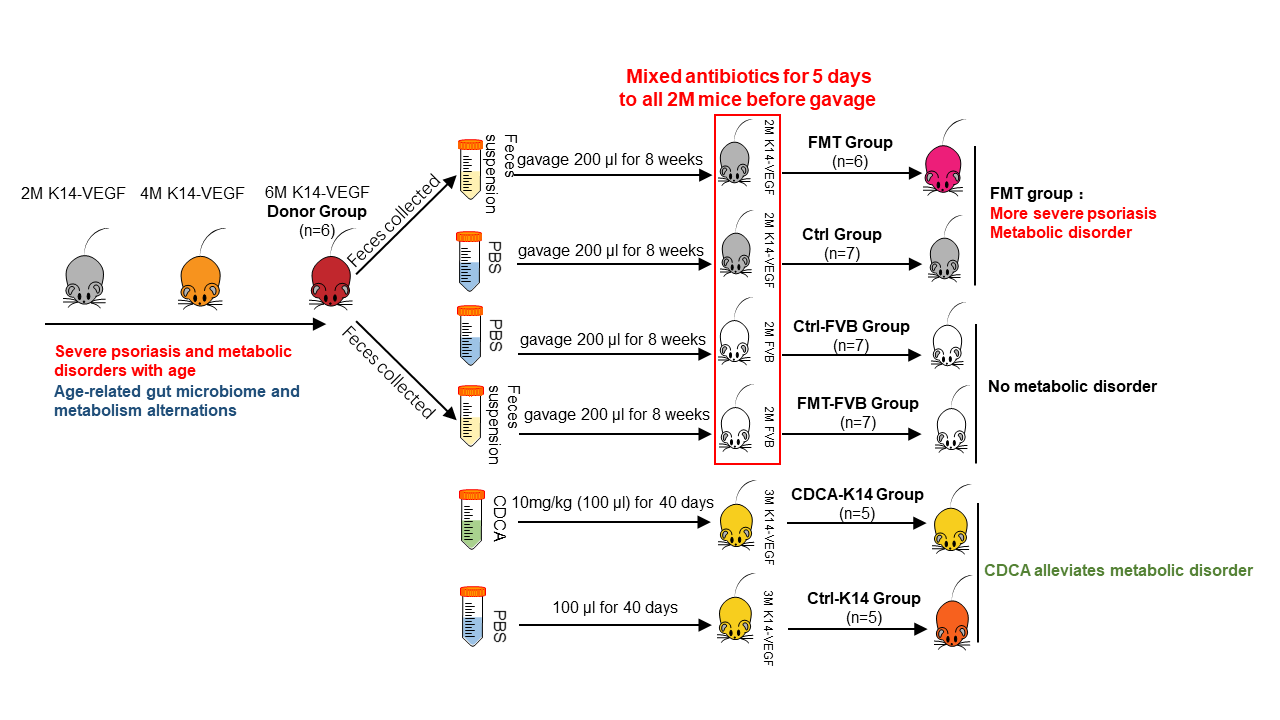


**Supplement FIGURE 1. A schematic representation of our study design.**

**
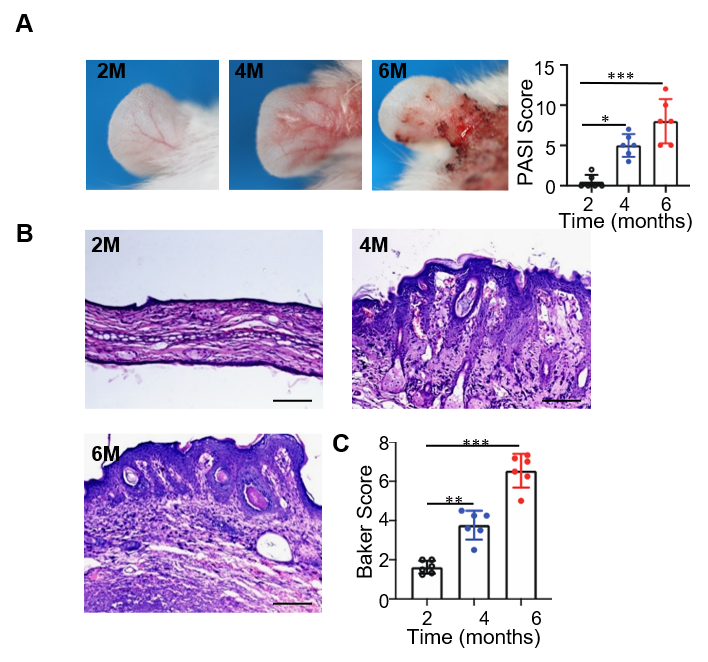
**

**Fig S2. The psoriatic phenotype of K14-VEGF-A transgenic mice exacerbated with age.**

**(A)**Representative ear images of 2,4 and 6-month K14-VEGF mice. PASI scores were depicted (right). **(B)**Representative H&E-stained ear sections from mice (n=6 per group). Scale bar, 100 mm. **(C)**Baker score of psoriasis severity.

Statistical analysis for **(B)** to **(C)**: each symbol represented a mouse; bars showed means and SEM. Statistical tests used were one-way ANOVA. *P < 0.05, **P < 0.01, and ***P < 0.001.


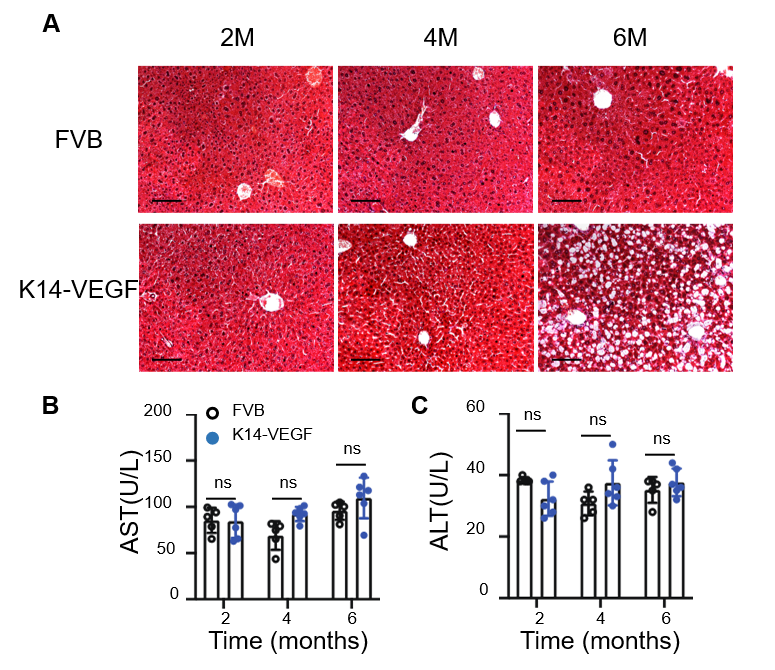


**Fig S3. K14-VEGF-A transgenic mice showed no signatures of liver fibrosis or hepatitis with age.**

**(A)**Masson-stained sections of liver from K14-VEGF mice at the age of 2,4 and 6 months (n=6 per group). Scale bar, 100 mm. **(B)**Serum ALT and **(C)**AST levels.

Statistical analysis for **(B)**: each symbol represented a mouse, bars showed means and SEM. Statistical tests used were Two-way ANOVA. *P < 0.05, **P < 0.01, and ***P < 0.001.


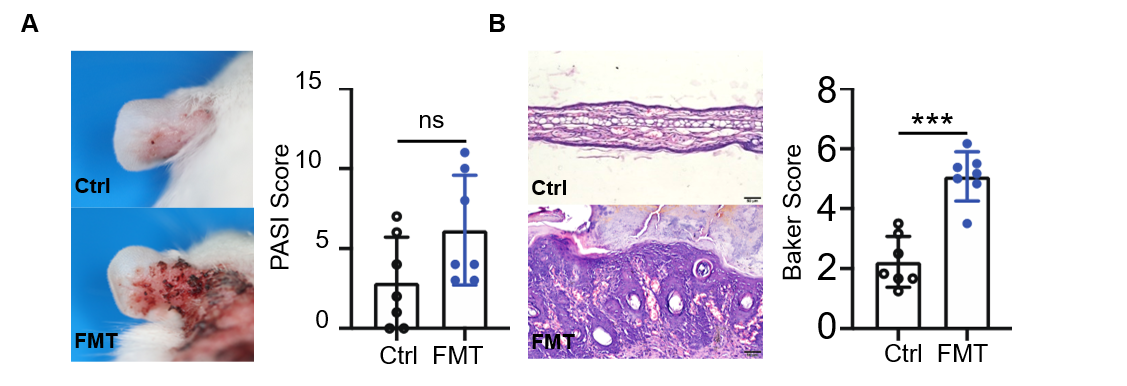


**Fig S4. Fecal transfer aggravated psoriasis of FMT group.**

**(A)**Representative ear images of FMT and Ctrl group. PASI scores were depicted (right). **(B)**Representative H&E-stained sections of FMT and Ctrl group. Scale bar, 100 mm. Baker score of psoriasis severity were depicted (right). Statistical analysis for **(A)** to **(B)**: each symbol represented a mouse; bars showed means and SEM. Statistical tests used Student t test. *P < 0.05, **P < 0.01, and ***P < 0.001.

**
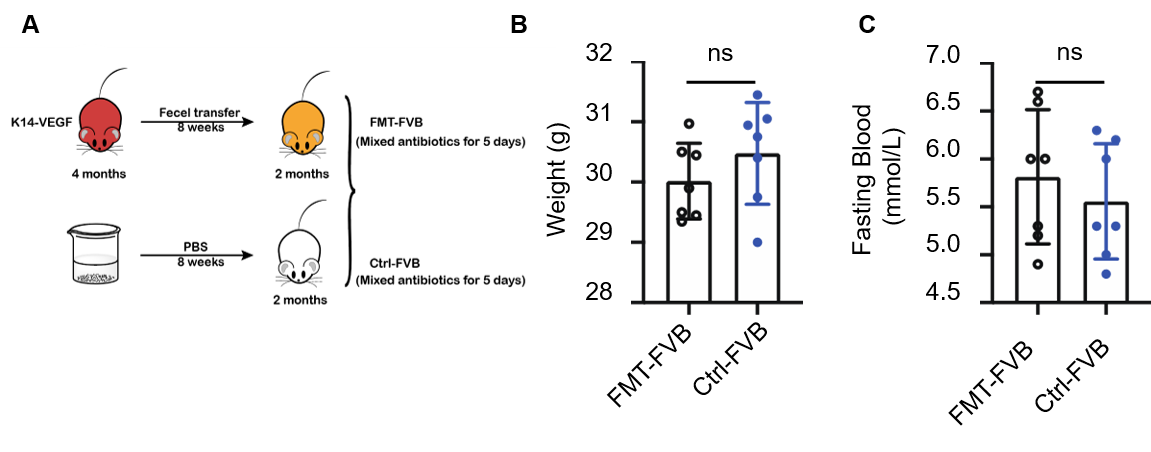
**

**Fig S5. Fecal transfer from K14-VEGF-A transgenic mice into FVB mice induced no metabolic disorder.**

**(A)**Fecal microbiota transplant experimental diagram. All 2-month FVB mice were treated with mixed antibiotic for 5 days, then transferred with feces of 6-month K14-VEGF mice as K14-FVB group, or PBS as K14-control group by gavage for 8 weeks. **(B)**Body weight of mice. **(C)**Fasting blood glucose levels. Statistical analysis for **(B)** to **(C):** each symbol represented a mouse; bars showed means and SEM. Statistical tests used Student t test. *P < 0.05, **P < 0.01, and ***P < 0.001.
